# Alkaline Extraction, Structural Characterization, and Bioactivities of (1→6)-β-d-Glucan from *Lentinus edodes*

**DOI:** 10.3390/molecules24081610

**Published:** 2019-04-24

**Authors:** Jia Li, Chao Cai, Mengmeng Zheng, Jiejie Hao, Ya Wang, Minghua Hu, Luodi Fan, Guangli Yu

**Affiliations:** 1 Key Laboratory of Marine Drugs of Ministry of Education & Shandong Provincial Key Laboratory of Glycoscience and Glycotechnology, School of Medicine and Pharmacy, Ocean University of China, Qingdao 266003, China; lijia199208@163.com (J.L.); 2009haojie@ouc.edu.cn (J.H.); yawangouc@163.com (Y.W.); 2 Laboratory for Marine Drugs and Bioproducts, Qingdao National Laboratory for Marine Science and Technology, Qingdao 266237, China; 3 Laboratory of Chinese Medicine Pharmacy, School of Pharmacy, Shandong University of Traditional Chinese Medicine, Jinan 250355, China; 18364166659@163.com; 4 Infinitus (China) Company Ltd., Guangzhou 510600, China; Mandy.Hu@infinitus-int.com (M.H.); Franz.Fan@infinitus-int.com (L.F.)

**Keywords:** *Lentinus edodes*, orthogonal optimization, (1→6)-β-d-glucan, structure characterization, solution conformation, antioxidant, immunomodulatory activities

## Abstract

The purpose of this study is to develop a robust approach to obtain β glucans from *Lentinus edodes* and to characterize their structural and biological properties for sustainable utilization. The alkali extraction was optimized with an orthogonal experimental design, and a concise process for obtaining specific targeting polysaccharides from *Lentinus edodes* was developed in this study. After purification with a Q-Sepharose Fast Flow strong anion-exchange column, the monosaccharide composition, a methylation analysis, and NMR spectroscopy were employed for their structural characterizations. LeP-N2 was found to be composed of (1→6)-β-d-glucans with minor β-(1→3) glucosidic side chains. Atomic force microscopy (AFM) and high-performance gel permeation chromatography–refractive index–multi-angle laser light scattering (HPGPC-RI-MALLS) also revealed LeP-N2 exhibiting a compact unit in aqueous solution. This (1→6)-β-d-glucan was tested for antioxidant activities with IC_50_ at 157 μg/mL. Moreover, RAW 264.7 macrophage activation indicated that the release of nitric oxide (NO) and reactive oxygen species (ROS) were markedly increased with no cytotoxicity at a dose of 100 μg/mL. These findings suggest that the (1→6)-β-d-glucans obtained from *Lentinus edodes* could serve as potential agents in the fields of functional foods or medicine.

## 1. Introduction

In recent years, polysaccharides have attracted considerable attention as bioactive ingredients and food additives in the medical and food industry [1,2]. The representative polysaccharides isolated from Chinese traditional herbs, including *Ganoderma lucidum*, *Cordyceps*, and *Lentinus edodes*, have been extensively studied due to their potent antitumor properties. The most common polysaccharides presented in mushrooms are glucans mainly composed of homo-/heterogeneous β-(1→3), β-(1→6), and α-(1→4) glycosides possessing antitumor and immunostimulating activities [3,4]. *Lentinus edodes (Berk.) sing*, as folk tonic foods and traditional Chinese medicines, have been widely applied in both food and the medical field for centuries in Asian countries due to their nutritional value and medicinal properties [5]. Lentinan (LNT), a typical β-glucan isolated from the fruiting body of *Lentinus edodes*, has been widely used in clinics as an antitumor drug since its first use in Japan in the early 1980s. It also possesses numerous bioactivities, such as anti-oxidation [6,7], antitumor activities [8,9], and immunomodulation [10,11]. In addition, due to its unique triple helical conformation, the antitumor activity of lentinan is exerted not by a direct cytotoxic effect on cancer cells but prevalently through the activation of the host immune system, inducing macrophage and T-cell synergistic antitumor activities [5,12].

The biological activities of polysaccharides are closely related to their structural characteristics, such as molecular weight, monosaccharide composition, α/β-configuration, conformation, glycosidic linkage, branching degree, and length of side chains [13,14,15]. Furthermore, the strain, origin, climate, as well as the extraction and purification procedure also affect the abovementioned characteristics [16,17].

Compared to the intensive research of β-(1→3)-glucans, relatively fewer studies have been done on the structural and immunomodulatory properties of β-(1→6)-glucans [18,19]. The influence of the chain conformation and molecular weight on the biological activities of the β-(1→6)-glucan has not yet been well-established due to the difficulty of preparing glucans with specific structural properties. Moreover, the structures of natural glucans are diverse, and the sustainable development of bioactive polysaccharides is of great interest to glycochemists. It is presumed that (1→6)-β-d-glucans are commonly found in the cell-surface lipopolysaccharides (LPSs) and capsular polysaccharides (CPSs). Jeff et al. reported a (1→6)-β-d-glucans (WPLE-N-1) from *Lentinus edodes* and showed the highest inhibition of tumor cell proliferation compared to mannogalactoglucans [20].

The present study reported herein aims to achieve the sustainable utilization of *Lentinus edodes*. The protocol developed in this study successfully separated β-glucan from the α/β glucan mixtures. A series of polysaccharides including a (1→6)-β-d-glucan were obtained using optimized alkaline extraction, ethanol precipitation, and Q-Sepharose Fast Flow purification. Their chemical and conformational structures were characterized by NMR (^1^H, ^13^C, ^1^H-^13^C HSQC and ^1^H-^1^H COSY) and a methylation analysis combined with a monosaccharide composition analysis. The molecular weights and chain conformations were determined with high-performance gel permeation chromatography–refractive index–multi-angle laser light scattering (HPGPC-RI-MALLS) and atomic force microscopy (AFM). In addition, bioactivities such as the hydroxyl radical scavenging activity and RAW 264.7 macrophage activation effects were carried out in vitro.

## 2. Results

### 2.1. Orthogonal Alkaline Extraction of Lentinus Edodes Polysaccharides

In this study, the primary factors for alkaline extraction of crude polysaccharides from *Lentinus edodes* were examined using an L_9_ (3^3^) experiment with an orthogonal design. The K and R values are listed in Table S1, and it was found that the factors affecting the extraction yields were in the following order: C (sodium hydroxide concentration) > B (extraction time) > A (extraction temperature). The concentration of sodium hydroxide (NaOH) exerted the most significant effect (*p* < 0.05) on the extraction yields of crude polysaccharide, and the optimum alkaline extraction condition was determined to be in the solution of NaOH at 0.5 mol/L at 60 °C for 2 h, which was according to the yield as an evaluation index.

The samples obtained from the orthogonal experiment (OE) were further characterized using ^1^H-NMR spectroscopy, as shown in Figure S1. Two anomeric proton signals at δ_H_ 4.24 and 5.07 ppm corresponded to the glucopyranosyl units with β- and α-configurations, respectively. Interestingly, it was found that OE-1, OE-6, and OE-8 extracted from 0.1 M NaOH all possessed relatively low α-configurations, whereas OE-3, OE-5, and OE-7 acquired from 0.5 M NaOH exhibited high α-configurations. These results show that the variable extraction condition could obtain glucans with α or β configurations separately. This efficient extraction and separation method provides support for the preparation of polysaccharides from *Lentinus edodes* with unique structures. We expected a method to obtain a high purity of β-glucans. Based on the above conclusions, we selected 0.1 M NaOH solution as the extraction solvent, and the optimization process was modified as extracted with 0.1 M NaOH at 60 °C for 2 h.

### 2.2. Purification and Chemical Properties of LeP-N2

**Figure 1:**
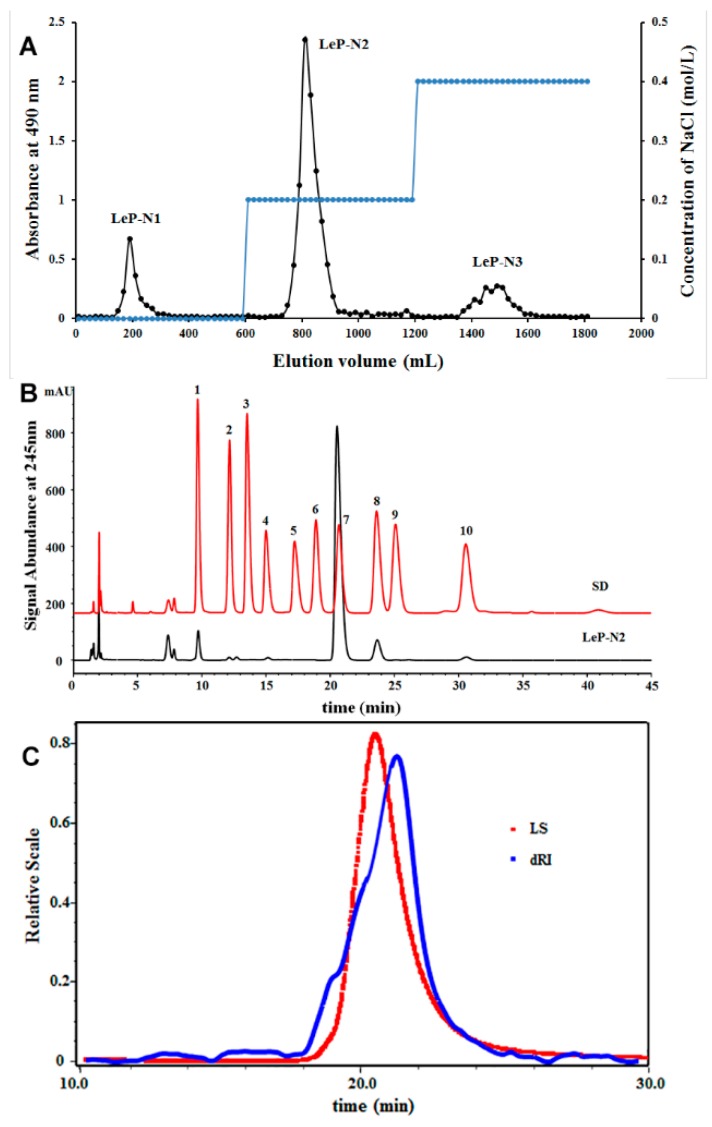
The purification and chemical properties of LeP-N2: (**A**) A stepwise elution curve of crude polysaccharide (LeP-N) from *Lentinus edodes* on a Q-Sepharose Fast Flow (QFF) strong anion-exchange column. (**B**) A high-performance liquid chromatography (HPLC) chromatogram of the monosaccharide composition (1. Man, 2. GlcN, 3. Rha, 4. GlcA, 5. GalA, 6. GalN, 7. Glc, 8. Gal, 9. Ara, and 10. Fuc). (**C**) The molecular weight distribution by HPGPC-RI-MALLS in a 0.1 M Na_2_SO_4_ aqueous solution.

The crude polysaccharides (LeP-N) was obtained by ethanol precipitation according to the modified optimized extraction conditions. In order to obtain a high-purity polysaccharide, LeP-N was loaded onto a Q-Sepharose Fast Flow (QFF) strong anion-exchange column and eluted with distilled water and 0.2 and 0.4 mol/L NaCl, respectively. Finally, three purified polysaccharides fractions (LeP-N1, LeP-N2, and LeP-N3) were obtained. As shown in Figure 1A, LeP-N2 was the main component of LeP-N with a high purity, which was chosen for further studies.

The phenol-sulfuric acid method showed that the total carbohydrates of LeP-N2 was 95.8%. Moreover, the protein contents of LeP-N2 was 1.1%. As shown in Figure 1B, based on the retention time and the peak area of each monosaccharide standard, the monosaccharide composition of LeP-N2 was then calculated. Approximately, 97.1% of glucose was content in LeP-N2, which indicated that a high purity and homogeneous structural glucans could be obtained through the alkaline extraction and QFF column purification. It was speculated that the alkaline solution could destroy and remove the proteins and other impurities after precipitation.

### 2.3. Structural Characterization of the β-Glucan LeP-N2

#### 2.3.1. Methylation Analysis

**Figure 2:**
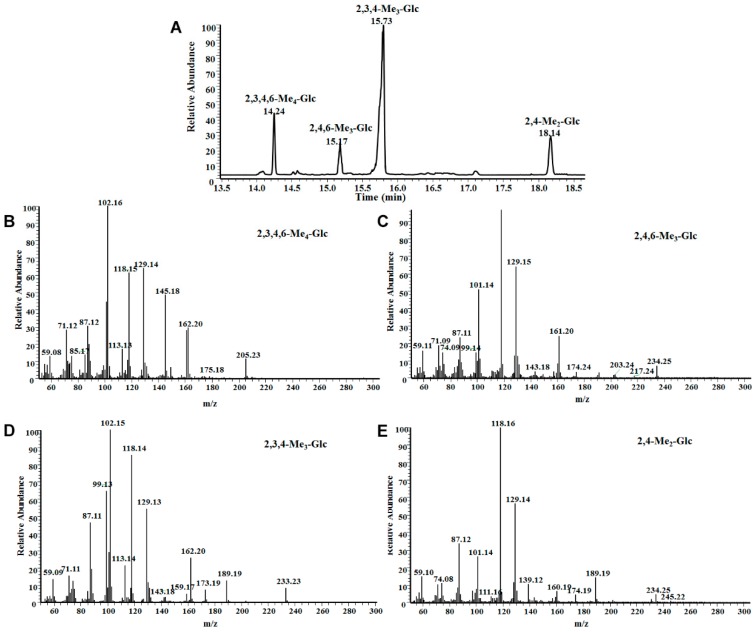
The GC-MS data of the partially methylated alditol acetates of LeP-N2. (**A**) GC-MS elution spectra of LeP-N2 after methylation. (**B**) Mass spectrometry fragments of Glc*p*-(1→. (**C**) Mass spectrometry fragments of →3)-Glc*p*-(1→. (**D**) Mass spectrometry fragments of →6)-Glc*p*-(1→. (**E**) Mass spectrometry fragments of →3, 6)-Glc*p*-(1→.

A methylation analysis is a technique widely used in the structural analysis of complex polysaccharides, indicating the types and linkages of glycosyl units. A full methylation was confirmed by the analysis of a FT-IR spectrum in Figure S2. The broad peaks at 3385 cm^−1^ corresponding to an O–H stretching vibration totally disappeared, indicating that the full methylation had been completed successfully. The GC-MS profile of partially methylated alditol acetates for LeP-N2 is shown in Figure 2.

According to the comparison with the Complex Carbohydrate Research Center (CCRC) spectral database, the partially methylated alditol acetates of LeP-N2 are shown in Figure 2. The results depicted in Table 1 show the presence of (1→6)-linked, (1→3)-linked, (1→3, 6)-linked, and terminal glucopyranosyl residues. The mole ratio of (1→6)-Glc*p* was as high as 63.1%, and (1→3)-Glc*p*, (1→3, 6)-Glc*p*, and →1)-Glc*p* were respectively approx. 10%, indicating that LeP-N2 was mainly composed of (1→6)-linked glucan with small amounts of the (1→3)-linked glycosidic side chain. A similar conclusion was confirmed through an NMR analysis. 

#### 2.3.2. NMR Analysis

**Figure 3:**
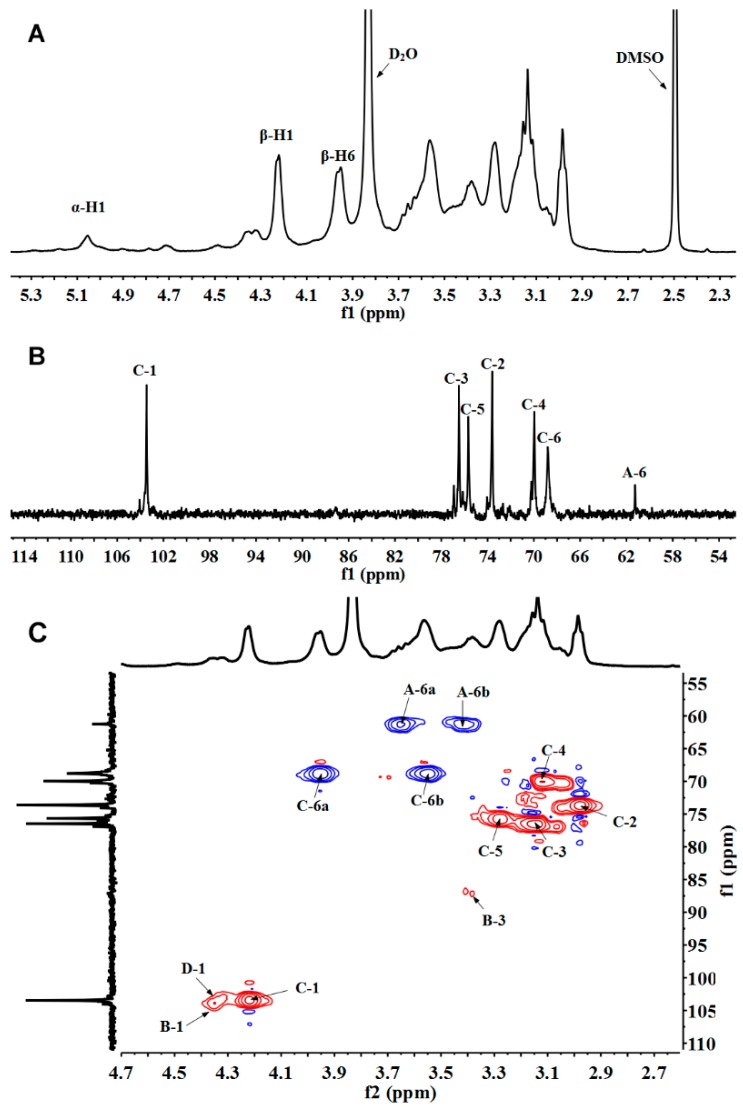
The NMR spectra of LeP-N2: (**A**) ^1^H NMR spectrum in DMSO/D_2_O (6:1) at 60 °C; (**B**) ^13^C NMR spectrum in DMSO/D_2_O (6:1) at 25 °C; and (**C**) ^1^H-^13^C HSQC spectrum spectra.

The ^1^H and ^13^C-NMR spectra of LeP-N2 was analyzed in DMSO/D_2_O (6:1 *v*/*v*). As shown in Figure 3, two anomeric proton signals at δ_H_ 4.22 and 5.06 ppm indicated the H-1 of β- and α-configuration glucopyranosyl units. The characteristic peak at δ_C_ 103.42 ppm indicated the signal of anomeric carbon on β- glucopyranosyl residues. The carbon signals of C-6 were downfield shifted to 68.82 ppm from δ_C_ 60.16 ppm due to the glycosidic linkages formed at position O-6 of β-glucan. The ^13^C NMR was highly consistent with β-(1→6)-d-glucans produced by strains of *Botryosphaeria rhodina* isolated from rotting tropical fruit reported by Vasconcelos et al. [21]. Correspondingly, other carbon signals that overlapped at δ_C_ 60.00–86.00 ppm were also well-signed according to 2-D NMR spectra. The cross-peaks of ^1^H and ^13^C of LeP-N2 could be further identified by ^1^H-^1^H COSY (Figure S3) and ^1^H-^13^C HSQC spectra (Figure 3C). The chemical shifts of residue C (1→6)-β-d-Glc*p* were assigned at δ 103.42/4.22, 73.69/2.98, 76.55/3.14, 70.02/3.12, 75.73/3.29, and 68.82/3.55, 3.95 ppm, corresponding to C1/H1, C2/H2, C3/H3, C4/H4, C5/H5, C6/H6a, and H6b. In addition, the signals at 104.08/4.36, 103.42/4.34, and 103.26/4.32 ppm were assigned to the anomeric signals of C1/H1 signals for residue A →1)-β-d-Glc*p*, residue B (1→3)-β-d-Glc*p*, and residue D (1→3,6)-β-d-Glc*p*, respectively. Therefore, all proton and carbon signals were assigned and summarized (Table 2).

### 2.4. Chain Conformation of the β-Glucan LeP-N2

#### 2.4.1. Molecular Weight and Solution Behavior

**Figure 4:**
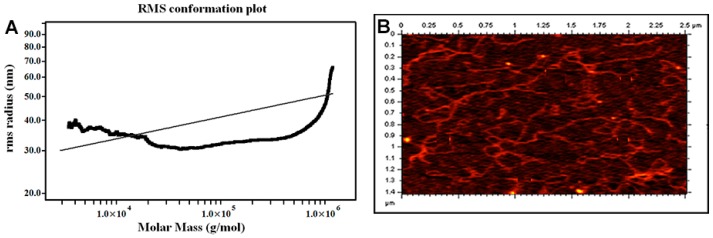
The chain conformation characterization of LeP-N2: (**A**) The plots of <S^2^>_z_^1/2^ vs. M_w_ in double logarithmic coordinates in a 0.1 M Na_2_SO_4_ aqueous solution at 35 °C and (**B**) atomic force microscopy (AFM) images in an aqueous solution.

In this study, the molecular weights and chain conformations of the purified glucans were analyzed with HPGPC-RI-MALLS. Figure 1C shows the molecular weight distribution of LeP-N2 in a 0.1 M Na_2_SO_4_ aqueous solution at 35 °C. Hence, the average molecular weights of LeP-N2 was calculated by the Astra Software 6.1.1 at approximately 96 kDa. In addition, the conformational and structural information, including <S^2^>_z_^1/2^ (RMS radius), polydispersity index (PDI, M_w_/M_n_), and the curve of <S^2^>_z_^1/2^–M_w_ were also carried out with HPGPC-RI-MALLS. The RMS radius and PDI of LeP-N2 was calculated to be 39.9 nm and 1.59, respectively. The double logarithmic relationship of <S^2^>_z_^1/2^-M_w_ of LeP-N2 obtained from an alkaline extraction in a 0.1 M Na_2_SO_4_ solution is depicted in Figure 4A. The β value of LeP-N2 was determined to be 0.26 in the range of 0.2–0.4, indicating a highly branched structure. 

#### 2.4.2. AFM Intuitive Display

To provide direct evidence of the chain conformation of polysaccharides, AFM was used to analyze their morphology. LeP-N2 possessed a high-branched chain structure in Figure 4B. The advanced morphological structures of polysaccharides were visually observed by AFM in an aqueous solution, which was also indicated by the abovementioned results. This provides a favorable technique for acquiring the structure–activity relationship (SAR) of the natural polysaccharides.

### 2.5. The Bioactivity Evaluation of the β-Glucan LeP-N2

#### 2.5.1. Assay of Hydroxyl Radical Scavenging Activity

**Figure 5:**
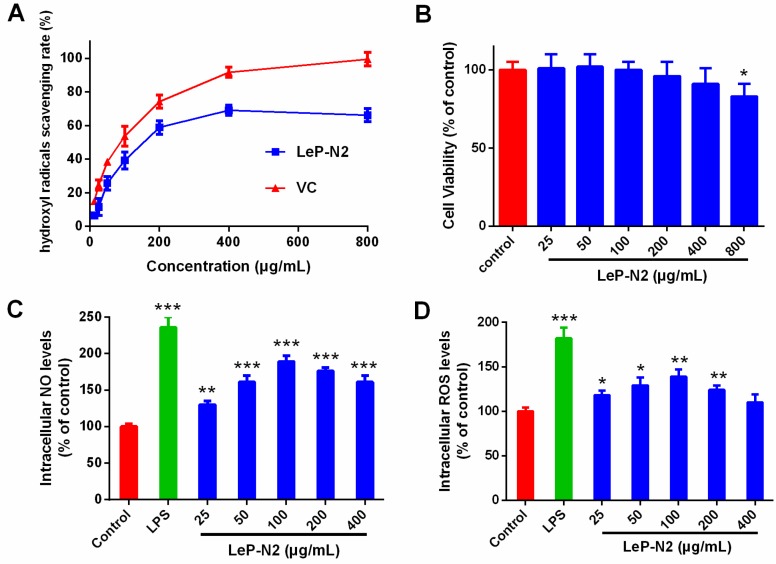
The bioactivities of LeP-N2: The effects on (**A**) the Hydroxyl radical scavenging activity; (**B**) the cell viability; and the (**C**) nitric oxide (NO) and (**D**) reactive oxygen species (ROS) production. The data are means ± SD of triplicate experiments. Significant differences between the groups were analyzed with a Students’ *t* test. * *p* < 0.05, ** *p* < 0.01, and *** *p* < 0.001 vs. the untreated group (medium).

Figure 5A shows the hydroxyl radical scavenging activity, which was dose-dependent at low concentrations. It was found that the scavenging activity decreased until more than 800 μg/mL, possibly due to the weak toxicity. The IC_50_ values of LeP-N2 was identified to be 157 μg/mL, which was comparable to 92 μg/mL of Vitamin C (VC). The present results indicate that LeP-N2 exhibits a good antioxidant activity with a dose-dependence at low concentrations.

#### 2.5.2. Effects on RAW 264.7 Macrophage Activation

The polysaccharides lentinan obtained from *Lentinus edodes* have been marketed for clinical application, and their characteristic structure represents a β-(1→3)-d-glucan with β-(1→6) glucosidic side branches. The polysaccharide reported in this study possess unique structural properties, and their immunomodulatory effects were investigated. Figure 5B shows that LeP-N2 was no cytotoxic to RAW 264.7 cells below 100 μg/mL. When the concentration was increased to more than 100 μg/mL, LeP-N2 showed an obvious cytotoxicity and cell death rate at 800 μg/mL (*p* < 0.001). 

A macrophage activation was determined by assessing the release of toxic molecules, such as NO and ROS. As shown in Figure 5C,D, RAW 264.7 cells treated with LeP-N2 exhibited a dose-dependency in NO and ROS production below 100 μg/mL. Furthermore, due to the cytotoxicity of polysaccharides, NO and ROS production were slightly reduced at concentrations above 100 μg/mL.

## 3. Discussion

Although the lentinan polysaccharides extracted from *Lentinus edodes* were commonly obtained by water extraction, their structural characteristics were significantly variable due to the diverse extraction processes [2]. According to the results reported in previous studies, the yields of these polysaccharides were as low as 0.12% via the conventional hot water extraction [22]. To make extensive use of *Lentinus edodes* with an improved extraction yield, an alkaline solution was employed to effectively obtain the pure polysaccharides in this study. A concise extraction and separation method provided the unique polysaccharides from *Lentinus edodes* in the α or β configurations separately. We found that a β-glucan could be obtained from the NaOH alkaline solution of 0.1 mol/L up to 0.5 mol/L, and this phenomenon has not been reported yet to the best of our knowledge. To obtain β-glucan with a high purity, the optimized extraction process was modified as 0.1 M NaOH at 60 °C for 2 h, and LeP-N2 was subsequently obtained after purification by a QFF anion-exchange column eluted with 0.2 mol/L NaCl, which was chosen for further studies.

According to the methylation and NMR analysis, polysaccharide LeP-N2 was summarized as a β-(1→6)-d-glucan with small amounts of (1→3)-β-d-glucosidic side chains. The structures of lentinan polysaccharides reported previously [23,24] are also varied due to the different extraction methods.

Although the structure of Lep-N2 was similar to the structure of the WPLE-N-1 polysaccharide reported by Jeff et al. in 2013 [20], a totally different strategy with the alkaline extraction to obtain β-glucan from *Lentinus edodes* is reported in this study. Meanwhile, our method can selectively obtain the (1→6)-β-d-glucan from α/β glucan mixtures, which has not been reported in previous literature. In addition, the molecular weight of (1→6)-β-d-glucan reported in our study is 96 kDa, which is much smaller than the 757.5 kDa of WPLE-N-1 in Jeff’s work. 

The chain conformations of purified glucans were analyzed with AFM and HPGPC-RI-MALLS. It is worth mentioning that the slope β of the <S^2^>_z_^1/2^–M_w_ curve is related to the chain conformation and solution behavior of the polysaccharides in the specific solvent [25]. In general, the values of slope β are in the range of 0.2–0.4 and 0.5–0.6, which represent branching polymers with compact coil and flexible linear form, respectively. In particular, the values of slope β at 0.3 and 1.0 indicate a sphere-like and rigid rod polymer chain conformation, respectively [26,27]. Wang’s group [28] revealed that the triple-helical lentinan polysaccharides in an aqueous solution predominantly exhibited as worm-like linear, circular, and crossover species, and the disentanglement and transition from the triple helix to single flexible chain occurred simultaneously at 130–145 °C. Zeng and Tang also reported that highly branched polymers tended to form a “U-shaped” curve in the plots of <S^2^>_z_^1/2^ –M_w_ [29,30]. The LeP-N2 polysaccharide developed in this study showed a “U-shaped” curve. Thus, we speculate that the conformation of LeP-N2 is a branching polymer with a compact coil shape.

Natural polysaccharides isolated from different types of mushrooms have been reported to exhibit free radical scavenging activities [31,32]. The hydroxyl radicals were most toxic among all ROS, so it is crucial to remove them for the necessary protection of biological systems [33].In this study, the (1→6)-β-d-glucan from *Lentinus edodes* were evaluated for scavenging hydroxyl radicals and activating RAW 264.7 murine macrophages to promote NO and ROS production. Our results suggested that LeP-N2 exhibited a good antioxidant activity, which is comparable to VC. Meanwhile, the release of NO and ROS during a RAW 264.7 macrophage activation was markedly increased with no cytotoxicity at a dose of 100 μg/mL. It has been reported that the level of anti-oxidation and reactive oxygen species is correlated well with the generation and malign transformation of cancer cells. Thus, if a compound can enhance the level of anti-oxidation and clear the reactive oxygen species in cancer cells, it may inhibit the tumor cells growth [34]. We believe that polysaccharides exert an immunological antitumor activity by clearing hydroxyl radicals in the normal environmental and by activating macrophages releasing ROS/NO in the tumor environment, which indicated that the (1→6)-β-d-glucan isolated from *Lentinus edodes* could serve as potential agents of functional foods or medicine.

## 4. Materials and Methods 

### 4.1. Materials

Trifluoroacetic acid (TFA), 1-phenyl-3-methyl-5-pyrazolone (PMP), phenol, sulphuric acid, alcohol, sodium borohydride (NaBH_4_), and methyl iodide (CH_3_I) were purchased from Sinopharm Chemical Reagent Co., Ltd. (Shanghai, China). Dimethyl sulfoxide (DMSO); DMSO-*d*_6_; and the monosaccharide standards mannose (Man), glucosamine (GlcN), rhamnose (Rha), glucuronic acid (GlcA), galacturonic acid (GalA), galactosamine (GalN), glucose (Glc), galactose (Gal), arabinose (Ara), and fucose (Fuc) were purchased from Sigma-Aldrich Co. (St. Louis, MO, USA). All other reagents used in this study were of analytical grade and purchased from commercial vendors.

*Lentinus edodes* (Berk.) *sing* were provided by INFINITUS Company (Guangzhou, China).

### 4.2. Isolation and Purification of Lentinus Edodes Polysaccharides

In this study, the dried fruiting body powder of *Lentinus edodes* was first extracted using water (20:1 *v*/*w*) at 25 °C for 5 h, and then, the residue was dried for further use. The alkaline extraction was accomplished with a sodium hydroxide (NaOH) solution at a ratio of 20:1 (*v*/*w*) with two replicates. The extraction experiment consisted of three factors and three levels, namely extraction temperatures of 0, 20, and 60 °C; extraction times of 0.5, 1, and 2 h; and then sodium hydroxide concentrations of 0.1, 0.25, and 0.5 mol/L. The range of factor levels was based on the results of the preliminary experiments. The experimental design is shown in Table S2. Briefly, alkaline-soluble crude polysaccharides were extracted from the residue that had been extracted by water. Then, the supernatant was neutralized, dialyzed, and precipitated in ethanol (1:4 *v*/*v*). The crude polysaccharide of L_9_ (3^3^) orthogonal experiment (OE) was labeled OE-1, OE-2, ···, and OE-9. Both the extraction rate of crude polysaccharides and monosaccharide composition was taken as the index. Extraction rate (%) = mass of crude polysaccharides/mass of fruit body extracted by water × 100 percent. The monosaccharide composition-determined method was referred to in Section 4.3 of this paper.

**Scheme 1:**
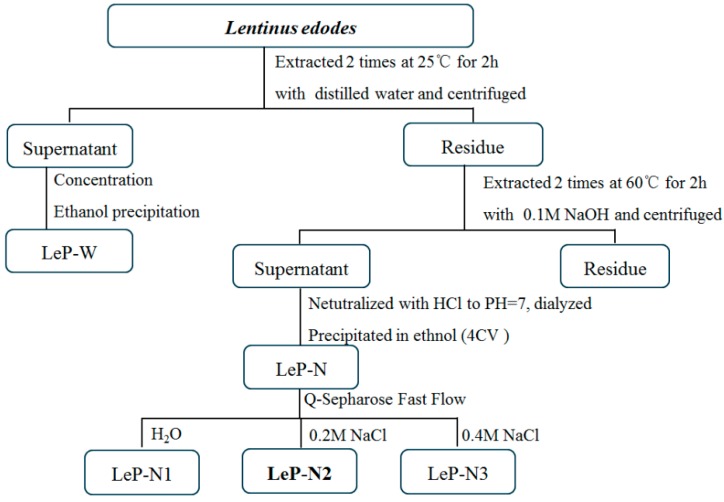
The scheme of extraction and purification of polysaccharides from *Lentinus edodes* (Berk.) *sing.*

In addition, the confirmation experiments were also carried out under modified optimized condition, as illustrated in the flowchart shown in Scheme 1. After freeze-drying, the crude polysaccharide of the confirmation experiments from *Lentinus edodes* were obtained and labeled as LeP-N.

LeP-N (200 mg) was dissolved in deionized water (15 mL), and the solution was subjected onto a Q-Sepharose Fast Flow strong anion-exchange column (QFF, 4.6 × 15 cm). The polysaccharide was eluted with 600 mL distilled water and 0.2 and 0.4 mol/L Sodium chloride (NaCl) solutions at a flow rate of 4 mL/min, respectively. Each 10 mL eluent was collected, and the polysaccharide was determined by the phenol-sulfuric acid method. Three fractions (LeP-N1, LeP-N2, and LeP-N3) were dialyzed and lyophilized. Through the structural characterization of three purified polysaccharide fractions, a β-d-glucan (LeP-N2) from *Lentinus edodes* was obtained and for further studies.

### 4.3. Chemical Properties and Monosaccharide Composition Analysis

The phenol-sulfuric acid method was employed to determine the total carbohydrates using d-glucose as a standard sample [35]. The protein content was evaluated using coomassie brilliant blue G-250, which was according to the Bradford Assay method [36].

The analysis of the monosaccharide composition was conducted according to the method described by Chen et al. [37], with slight modifications. Briefly, the polysaccharide sample was hydrolyzed with 4 M TFA at 110 °C for 6 h. Then, methanol was added and fully evaporated three times. The samples and monosaccharide standard mixture were mixed with a 0.3 M NaOH and PMP solution (dissolved in methanol) and incubated at 70 °C for 1 h. The reaction was stopped by neutralization with 0.3 M hydrochloric acid (HCl) and extracted with 0.5 mL of chloroform five times. The final aqueous layer was collected and analyzed using high-performance liquid chromatography (HPLC, Agilent1260, Agilent Technologies, Inc., Santa Clara, CA, USA). The monosaccharide standards (Man, GlcN, Rha, GlcA, GalA, GalN, Glc, Gal, Ara, and Fuc) were used to qualify the monosaccharide compositions.

### 4.4. Methylation Analysis of Lentinus Edodes Polysaccharides

According to the method of Anumula [38], different residues with slightly modified linkage types were determined via a methylation analysis. Briefly, the polysaccharide samples were dissolved in DMSO and methylated with sodium hydride (NaH) and methyl iodide (CH_3_I) for 3 h. The reaction was terminated with water and extracted three times with chloroform. After methylation, the chloroform layers were concentrated under a reduced pressure and analyzed by infrared (IR) spectroscopy. The disappearance of the broad O–H stretching vibration at approximately 3385 cm^−1^ indicated that a full methylation was complete, and if not, the full methylation steps were repeated. The complete methylated product was added to 2 M TFA and degraded at 110 °C for 4 h and then reduced with 0.1 M NaOH (containing 0.48 M NaBD_4_) at room temperature for 4 h. The reduced sample was added to anhydrous pyridine and acetic anhydride for acetylation. The resulting partially methylated alditol acetates (PMAAs) were then determined by gas chromatography-mass spectrometer (GC-MS), and the data analysis employed the CCRC database [39]. 

### 4.5. Nuclear Magnetic Resonance (NMR) Spectroscopy

The freeze-dried polysaccharide was exchanged with deuterium by lyophilizing with D_2_O (99.96%, Sigma-Aldrich, Inc., St. Louis, MO, USA) three times. The samples were dissolved in a mixed solvent of DMSO-*d*_6_/D_2_O (6:1 *v*/*v*) to acquire a ^1^H NMR spectrum at 60 °C and a ^13^C NMR spectrum at 25 °C. The ^1^H, ^13^C, ^1^H-^1^H COSY, ^1^H-^13^C HSQC, and HMBC NMR spectra were obtained using an Agilent DD2 500 MHz spectrometer (Agilent, USA). 

### 4.6. Analysis of Molecular Weights and Chain Conformations

The molecular weight (M_w_) and chain conformation parameters of the polysaccharide samples were determined using high-performance gel permeation chromatography–refractive index–multi-angle laser light scattering (HPGPC-RI-MALLS). The testing conditions were performed essentially as described by Zhang et al. [40]. The molecular weight for the polysaccharide fractions was measured on a DAWN HELEOS-II laser photometer (Wyatt Technology Co., Santa Barbara, CA, USA). The samples were separated on a Shodex OHpak SB-804/803 HQ column (8.0 mm × 300 mm, 6 μm, Showa Denko, Tokyo, Japan) and eluted with a 0.1 M Na_2_SO_4_ solution at a flow rate of 0.6 mL/min. The dn/dc value of the samples in a 0.1 M Na_2_SO_4_ solution were to be 0.135 mL/g at 658 nm. The data were analyzed using the Wyatt ASTRA v 6.1.1 software.

### 4.7. Atomic Force Microscope (AFM) Visualization

AFM imaging was conducted with an Agilent 5100 atomic force microscope (Agilent Co., USA). The polysaccharides were dissolved in deionized water and gradually diluted to 1 μg/mL. After being filtered, 1 μL of the sample solution was dripped onto the mica surface substrate, which was pretreated with adhesive tape and vacuum-dried for 5 h. The prepared sample was then imaged under a tapping mode atomic force microscope (TM-AFM) in a constant temperature and pressure operation box.

### 4.8. Hydroxyl Radical Scavenging Activity

The hydroxyl radical (·OH)-scavenging activity was performed according to the procedure previously described by Zhao et al. [41]. In brief, a FeSO_4_ solution (9 mmol/L), polysaccharides of different concentrations in aqueous solution, and H_2_O_2_ (8.8 mmol/L) were added in order. After incubation for 10 min, a salicylic acid solution (9 mmol/L) was added. Thirty minutes later, the absorbance was measured at 510 nm. The scavenging activity was calculated using the following equation:

Scavenging activity (%) = {(A_0_ − A_1_)/A_0_} × 100

where A_0_ is the absorbance value of the blank control and A_1_ is the absorbance of a certain concentration of polysaccharide or VC.

### 4.9. RAW264.7 Cell Line Culture

RAW264.7 macrophages were purchased from American Type Culture Collection (Manassas, VA, USA). The cells were maintained in an Roswell Park Memorial Institute (RPMI) 1640 medium supplemented with 10% (*v*/*v*) fetal bovine serum, penicillin (100 U/mL), and streptomycin (100 μg/mL) at 37 °C in a humidified incubator with 5% carbon dioxide (CO_2_). Before plating, the cells were dislodged from the plate by buting pipetting.

### 4.10. Cell Viability Assay

The cell viability was assessed using an MTT assay. The RAW 264.7 (5 × 10^3^ cells/well) were first treated with different concentrations of obtained polysaccharide samples and lentinan for 24 h, and their effects on the proliferation of RAW 264.7 were then detected with Cell Counting Kit 8 (CCK-8) (Seven Sea Pharmatech Co., Ltd., Beijing, China) according to the manufacturer’s protocol. The absorbance was measured at 450 nm on a BioRad model 680 Microplate Reader (Bio-Rad Laboratories, Inc., Hercules, CA, USA). The experiments were performed in triplicate and repeated at least three times. Cell proliferation was expressed as a percentage of the control set at 100 percent.

### 4.11. Nitric Oxide (NO) and Reactive Oxygen Species (ROS) Assays

The measurement of NO production and ROS was performed according to previous methods described by Yang et al. [42]. Briefly, cells were treated with LPS (1 μg/mL), lentinan, and different concentrations of LeP-N2. After incubation for 24 h, the supernatants were collected to determine the levels of NO and ROS using commercial kits according to the manufacturer’s instructions. The production of NO and ROS was expressed as a percentage of the control set to 100 percent.

## 5. Conclusions

In this study, the optimized extraction of polysaccharides from *Lentinus edodes* was determined through an orthogonal design with an alkaline solution (NaOH, 0.1 mol/L) at 60 °C for 2 h. It was also observed that a high concentration of alkaline is preferable for generating high levels of α-glucans. Under the optimized extraction condition and a purification through an QFF anion-exchange column, a (1→6)-β-d-glucan with (LeP-N2) was successfully obtained with a molecular weight of 96 kDa in a high purity. A methylation analysis and NMR spectroscopy were further employed to characterize the structure of polysaccharides isolated from *Lentinus edodes*, indicating the (1→6)-β-d-glucan main chain and (1→3)-β-d-glycosidic side chain. AFM and HPGPC-RI -MALLS revealed a compact unit with a “U-shaped” curve, representing a polysaccharide with a highly branched structure. In addition, LeP-N2 exhibited a good antioxidant activity. Meanwhile, LeP-N2 not only increased NO and ROS release by RAW 264.7 macrophages but also showed no cytotoxicity at low doses (<100 μg/mL). The preliminary study of biological activity suggests that this β-d-glucan purified from *Lentinus edodes* could be explored as potential natural antioxidant and immunostimulating agents for use in functional foods or medicine.

## Figures and Tables

**Table 1:** The GC-MS data of the main alditol acetate derivatives from methylated products of LeP-N2.

Methylated Alditol Acetates Derivative ^a^	Retention Time (min)	Type of Linkage	Relative Abundance (%)	Mass Fragments (*m*/*z*) (Relative Abundance, %)
2,3,4,6-Me_4_-Glc	14.2	Glc*p*-(1→	13.2	59,71,87,102,118,129,145,162,175,205
2,4,6-Me_3_-Glc	15.1	→3)-Glc*p*-(1→	10.9	59,71,87,101,118,129, 161,174,191,234
2,3,4-Me_3_-Glc	15.7	→6)-Glc*p*-(1→	63.1	59,71,87,99,102,118,129,162, 189,233
2,4-Me_2_-Glc	18.1	→3,6)-Glc*p*-(1→	12.8	59,74,87,101,118,129,139,160,189,234

^a^ 2,3,4,6-Me_4_-Glc*p* = 1,5-di-O-acetyl-2,3,4,6-tetra-O-methyl-Glucopyranose, etc.

**Table 2:** The ^13^C and ^1^H NMR chemical shifts of the main residues from LeP-N2 in D_2_O/DMSO (1:6 *v*/*v*).

Residues	δ^13^C ^a^/^1^H ^b^ (ppm)					
	C1/H1	C2/H2	C3/H3	C4/H4	C5/H5	C6/H6
A β-d-Glc*p* (→1	104.08/4.36	73.72/3.06	76.55/3.17	70.02/3.01	76.42/3.16	60.85/3.42, 3.65
B →3)-β-d-Glc*p*-(1→	103.42/4.34	73.09/3.19	87.25/3.35	68.9/3.15	76.55/3.11	59.73/3.35, 3.48
C →6)-β-d-Glc*p*(1→	103.42/4.22	73.69/2.98	76.55/3.14	70.02/3.12	75.73/3.29	68.82/3.55, 3.95
D →3, 6)-β-d-Glc*p*-(1→	103.26/4.32	72.91/3.20	87.1/3.39	68.26/3.27	75.73/3.30	68.82/3.55, 3.95

^a^ The values of the ^13^C chemical shifts were recorded with respect to the Me_2_SO as an internal standard and fixed at δ_C_ 39.339 ppm at 25 °C. ^b^ The values of the ^1^H chemical shifts were recorded with respect to the Me_2_SO as an internal standard and fixed at δ_H_ 2.494 ppm at 60 °C.

## Supplementary Materials

The following are available online, Table S1: The results of L_9_ (3^3^) orthogonal test for lentinus edodes polysaccharide. Table S2: Orthogonal extraction design of *lentinus edodes* polysaccharides. Figure S1: The ^1^H NMR spectrum of OEs in DMSO/D_2_O (6:1 *v*/*v*) at 60 °C. Figure S2: FT-IR spectrum of LeP-N2 after full methylation. Figure S3: The ^1^H-^1^H COSY spectrum of LeP-N2.
