# Supplementary material for: Alkaline Extraction, Structural Characterization, and Bioactivities of (1→6)-β-d-Glucan from Lentinus edodes
Source: Molecules. 2019 Apr 24;24(8):1610. doi: 10.3390/molecules24081610 (PMC6515283; doi:10.3390/molecules24081610)
Supplement: Supplementary file 1 [file molecules-24-01610-s001.pdf]

molecules

MDPI

Article

Alkaline extraction, structural characterization, and bioactivities of novel (1→6)-β-D-glucan from *Lentinus edodes*

Jia Li [1], Chao Cai [1,2,*], Mengmeng Zheng [3], Jiejie Hao [1,2], Ya Wang [1], Minghua Hu [4], Luodi Fan [4], Guangli Yu [1,2,*]

[1] Key Laboratory of Marine Drugs of Ministry of Education & Shandong Provincial Key Laboratory of Glycoscience and Glycotechnology, School of Medicine and Pharmacy, Ocean University of China, Qingdao 266003, China.; lijia199208@163.com (J. Li); caic@ouc.edu.cn (C. Cai); 18364166659@163.com (M. Zheng); 2009haojie@ouc.edu.cn (J. Hao); yawangouc@163.com (Y. Wang); Mandy.Hu@infinitus-int.com (M. Hu); Franz.Fan@infinitus-int.com (L. Fan); glyu@ouc.edu.cn (G.Yu)

[2] Laboratory for Marine Drugs and Bioproducts, Qingdao National Laboratory for Marine Science and Technology, Qingdao 266237, China., School of Medicine and Pharmacy, Ocean University of China, Qingdao 266003, China.

[3] Laboratory of Chinese medicine pharmacy, School of Pharmacy, Shandong University of Traditional Chinese Medicine, Jinan, 250355, China.

[4] Infinite Pole (China) Co., LTD., Guangdong, Guangzhou 510600, China

* Correspondence: caic@ouc.edu.cn (C. Cai); glyu@ouc.edu.cn (G.Yu) Tel.: +86-532-8203-1609

Contents

Table S1. The results of L_9 (3^3) orthogonal test for *lentinus edodes* polysaccharide

Table S2. Orthogonal extraction design of *lentinus edodes* polysaccharides

Figure S1. The ^1H NMR spectrum of OEs in DMSO/D_2O (6:1 v/v) at 60 ℃.

Figure S2. FT-IR spectrum of LeP-N2 after full methylation.

Figure S3. The ^1H-^1H COSY spectrum of LeP-N2

Table S1. The results of L_9 (3^3) orthogonal test

Orthogonal Experiment number	Factor			Extraction yield(%)
	Temperature A（℃）	Time B（h）	Concentration C（mol/L）	
1	0	0.5	0.1	3.59
2	0	1	0.25	6.11
3	0	2	0.5	9.84
4	20	0.5	0.25	5.98
5	20	1	0.5	9.50
6	20	2	0.1	5.28
7	60	0.5	0.5	8.95
8	60	1	0.1	7.11
9	60	2	0.25	7.73
K_1	6.513	6.173	5.327	6.940
K_2	6.920	7.573	6.607	6.780
K_3	7,930	7.617	9.430	7.643
R	1.417	1.444	4.103	

Table S2. Orthogonal extraction design of *lentinus edodes* polysaccharides

Level	Factor		
	Temperature A（℃）	Time B（h）	Concentration C（mol/L）
1	0	0.5	0.1
2	20	1	0.25
3	60	2	0.5

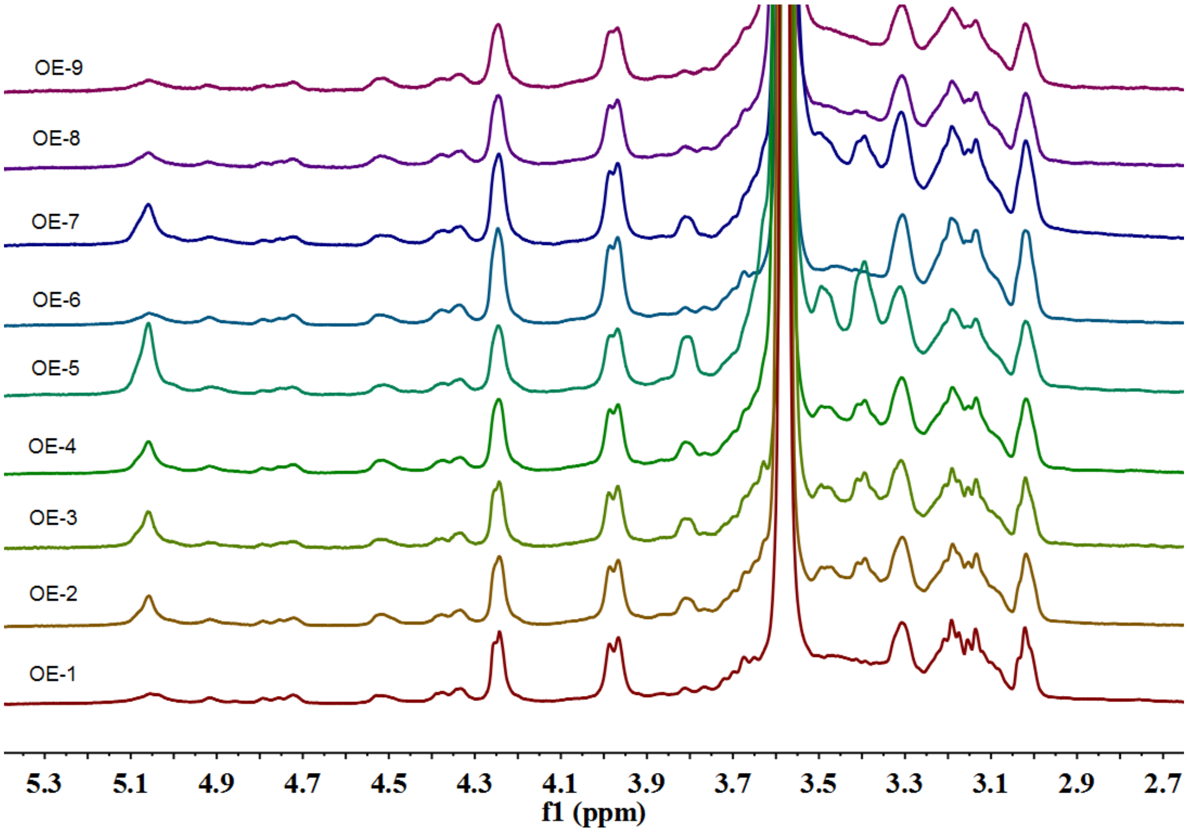

Figure S1. The ^{1}H-NMR spectrum of OEs in DMSO/D_2O (6:1 v/v) at 60℃.

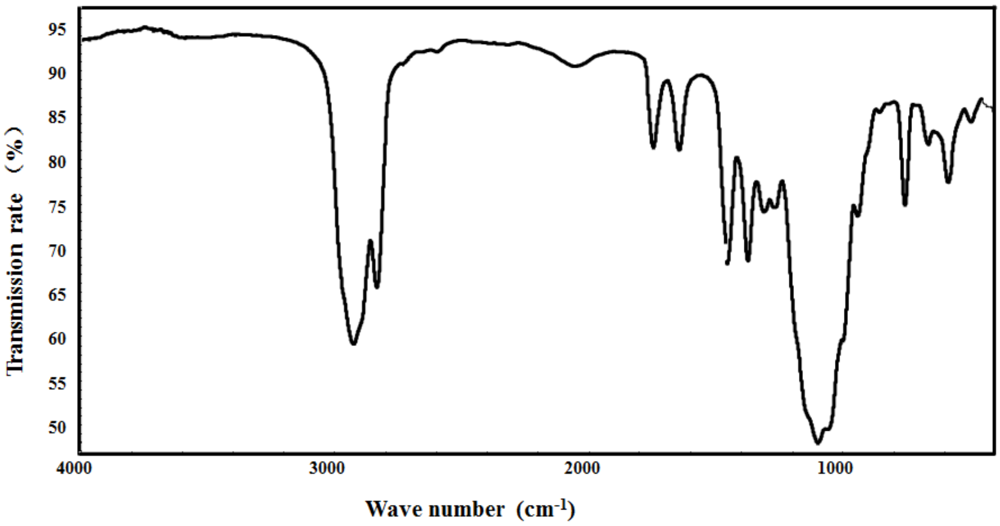

Figure S2. FT-IR spectrum of LeP-N2 after full methylation.

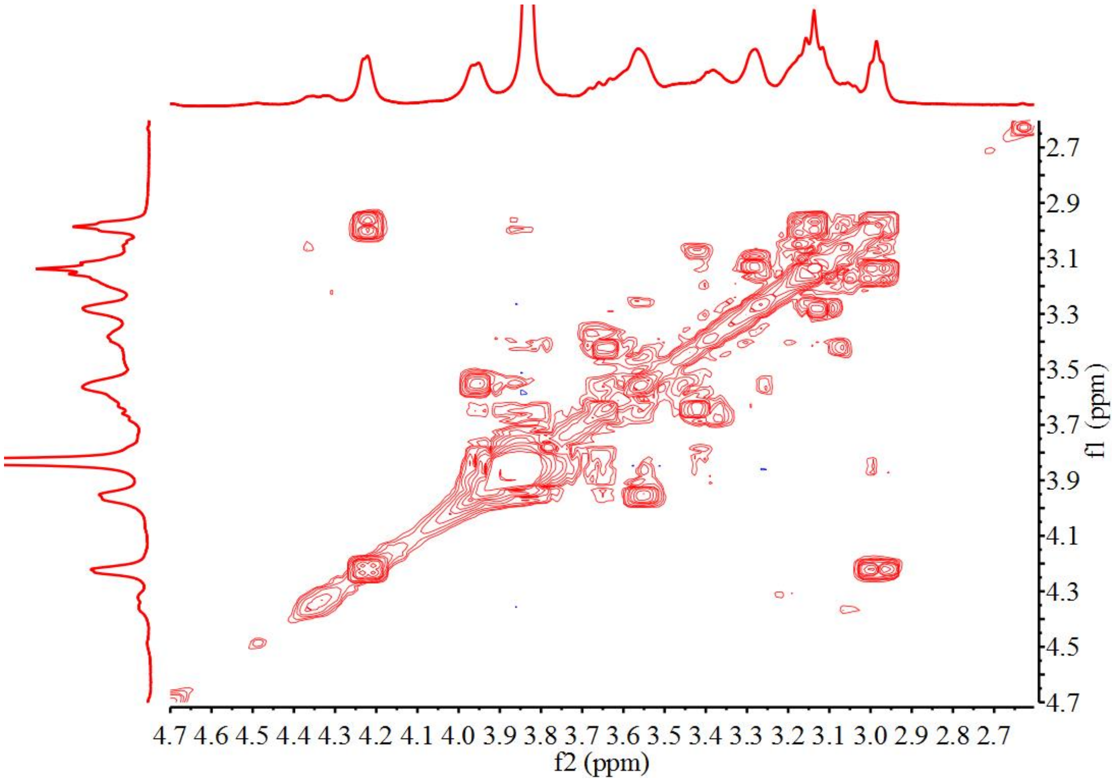

Figure S3. The ^{1}H-^{1}H COSY spectrum of LeP-N2
